# Insights Into Auditory Cortex Dynamics From Non-invasive Brain Stimulation

**DOI:** 10.3389/fnins.2018.00469

**Published:** 2018-07-13

**Authors:** Jamila Andoh, Reiko Matsushita, Robert J. Zatorre

**Affiliations:** ^1^Department of Cognitive and Clinical Neuroscience, Central Institute of Mental Health, Medical Faculty Mannheim, Heidelberg University, Mannheim, Germany; ^2^Montreal Neurological Institute, McGill University, Montreal, QC, Canada; ^3^International Laboratory for Brain, Music, and Sound Research (BRAMS), Montreal, QC, Canada

**Keywords:** non-invasive brain stimulation, auditory cortex, networks dynamics, asymmetry, interhemispheric interactions

## Abstract

Non-invasive brain stimulation (NIBS) has been widely used as a research tool to modulate cortical excitability of motor as well as non-motor areas, including auditory or language-related areas. NIBS, especially transcranial magnetic stimulation (TMS) and transcranial direct current stimulation, have also been used in clinical settings, with however variable therapeutic outcome, highlighting the need to better understand the mechanisms underlying NIBS techniques. TMS was initially used to address causality between specific brain areas and related behavior, such as language production, providing non-invasive alternatives to lesion studies. Recent literature however suggests that the relationship is not as straightforward as originally thought, and that TMS can show both linear and non-linear modulation of brain responses, highlighting complex network dynamics. In particular, in the last decade, NIBS studies have enabled further advances in our understanding of auditory processing and its underlying functional organization. For instance, NIBS studies showed that even when only one auditory cortex is stimulated unilaterally, bilateral modulation may result, thereby highlighting the influence of functional connectivity between auditory cortices. Additional neuromodulation techniques such as transcranial alternating current stimulation or transcranial random noise stimulation have been used to target frequency-specific neural oscillations of the auditory cortex, thereby providing further insight into modulation of auditory functions. All these NIBS techniques offer different perspectives into the function and organization of auditory cortex. However, further research should be carried out to assess the mode of action and long-term effects of NIBS to optimize their use in clinical settings.

## Introduction

Non-invasive brain stimulation (NIBS) has recently seen a surge of interest to provide understanding about the functional properties and interactions of cortical systems. With their ability to either inhibit or enhance cortical excitability, NIBS techniques are a promising tool in both research and clinical settings. Various types of NIBS are available: transcranial direct current stimulation (tDCS), transcranial magnetic stimulation (TMS), transcranial alternating current stimulation (tACS), or transcranial random noise stimulation (tRNS), which differ in their mode of action.

Here, we will review outcomes from NIBS studies in the context of their application to the auditory cortex and related systems. We will especially discuss how different NIBS techniques provide us with different perspectives in examining auditory processing, ranging from behavior to neural activity and structural organization. In addition, we will review how NIBS can be used to inform about the neural dynamics of auditory processing at basic processing levels, such as pitch, to higher order levels such as those involving speech. Finally, we discuss the potential of NIBS as a treatment tool for auditory-related disorders and highlight the need of basic research to increase our understanding of NIBS mechanisms.

## tDCS Modulation of the Auditory Cortex: Changes in Excitability and Behavior

Some of the first NIBS studies to investigate auditory processing used tDCS and assessed its effects on behavior (Mathys et al., 2010; Tang and Hammond, 2013; Matsushita et al., 2015). tDCS is a technique consisting of the delivery of low, constant electric currents transcranially on the cortical surface, thereby resulting in the modulation of cortical excitability. Standard tDCS uses two sponge electrodes [typically 5 cm × 5 cm or more, of opposite polarity: positive electrode (anode) and a negative electrode (cathode); DaSilva et al., 2011]. tDCS-induced effects depend on stimulation parameters such as stimulation polarity (anode, cathode) and timing of application (online, offline) and are described in the next section.

The role of tDCS polarity was shown over the motor cortex, with positive stimulation (anodal tDCS) increasing neuronal excitability (Nitsche and Paulus, 2000) and negative stimulation (cathodal tDCS) inhibiting it (Nitsche and Paulus, 2000; Nitsche et al., 2003b; Antal et al., 2004). The mechanisms of tDCS have been associated with long-term potentiation and long-term depression (LTP/LTD)-like plasticity (Nitsche et al., 2008; Fritsch et al., 2010; Monte-Silva et al., 2013). Moreover, when applied for a critical period of time, tDCS produces after-effects on cortical excitability which can last longer than 1 h (Bindman et al., 1964).

Transcranial direct current stimulation polarity-specific effects on auditory cortex are less clear. For instance, Zaehle et al. (2011) reported increases in the amplitude of auditory evoked potentials during a passive listening task following anodal stimulation (1.25 mA, 11 min) over the left temporal cortex compared with sham, but cathodal stimulation did not differ from sham stimulation. Additional factors to take into account when comparing tDCS applied over motor or auditory cortices relate to the excitability state of the targeted structures. Motor cortex is usually stimulated at rest, whereas sensory cortices are usually stimulated during relevant tasks, which might reverse the typical relationship between polarity of current flow and excitability (Filmer et al., 2014).

Changes in tDCS-induced cortical excitability depend also on the stimulation timing. In offline tDCS, stimulation is applied at rest, usually before the task is undertaken; whereas in online tDCS, stimulation is applied while a task is being executed. It is also common to use a combination of online and offline tDCS. The latter approach can have several variants, for example, tDCS and task stimuli start at the same time (Cohen Kadosh et al., 2010; Iuculano and Cohen Kadosh, 2013), or the task/stimuli start a few minutes after tDCS, and can continue after tDCS has ended (Stagg et al., 2011). Such procedure also enables to quantify the duration of tDCS-induced effects.

In Mathys et al. (2010), offline tDCS was applied over left and right temporal cortices in different sessions before the administration of a pitch discrimination task. The authors showed that anodal tDCS (2 mA, 25 min) had no effect, whereas cathodal tDCS impaired pitch discrimination performance, with a stronger impairment for the right temporal cortex stimulation. By contrast, using online tDCS during a pitch discrimination task, Tang and Hammond (2013) showed that anodal tDCS (1 mA, 20 min) applied over the right auditory cortex impaired performance. These authors however did not assess the effects of cathodal tDCS nor of stimulation on the left side. Using similar online tDCS parameters to the Tang’s study (i.e., 1 mA, 20 min), Matsushita et al. (2015) reported similar findings, such that anodal tDCS applied over the right auditory cortex disrupted auditory pitch learning compared to sham or left-auditory cortex tDCS. In addition, these authors found no significant differences between sham and cathodal tDCS (both stimulation types showed normal learning).

The different outcomes of the Matsushita and Tang’s vs. Mathys’ studies could be related to the stimulation timing (i.e., online vs. offline) relative to the task being performed or to the use of different stimulation parameters (intensity 2 vs. 1 mA, duration: 25 vs. 20 min). It has indeed been shown that 1-mA intensity caused conventional polarity-specific modulation of neural excitability (i.e., decreased for cathodal and increased for anodal), whereas 2 mA led to increased excitability from both stimulation polarities (Batsikadze et al., 2013). This could possibly explain that the Matsushita/Tang’s and Mathys’ studies showed impairment of pitch discrimination with anodal and cathodal tDCS, respectively. Moreover, the role of stimulation polarity for online vs. offline stimulation might underlie different neurobiological mechanisms, and deserves further investigation.

## Evidence of Lateralized Functions in the Auditory Cortex: Evidence From Neuroimaging and tDCS

Another issue which is not well understood is the asymmetry of tDCS-related effects in the auditory cortex. Neuroimaging findings for functional asymmetries in auditory cortices have been frequently reported, but there is still debate about their nature and whether they are directly or indirectly related to structural specialization (Dehaene-Lambertz et al., 2006). For instance, the role of left vs. right auditory cortices for processing speech or auditory stimuli was debated for some time (Zatorre and Gandour, 2008) and was discussed in the context of various acoustic features such as temporal or spectral information (Schonwiesner et al., 2005). Several authors have argued that the left auditory cortex seems to be specialized for rapid temporal processing, whereas the right auditory cortex might be involved in processing of fine spectral information (Belin et al., 1998; Zatorre and Belin, 2001; Zaehle et al., 2004, 2009). These functional asymmetries have been related to cortical structure of auditory cortex. For example, leftward asymmetry was reported for Heschl’s gyrus for cortical volume and cortical surface area (Penhune et al., 1996; Meyer et al., 2014), which was discussed in the context of a left auditory cortex preference for rapidly changing cues in spoken language signals (Poeppel, 2003). The asymmetry in volume of Heschl’s gyrus (Warrier et al., 2009) was also associated with functional neural activity in spectral and temporal tasks (Schonwiesner et al., 2005). Cortical thickness of Heschl’s gyrus was shown to be increased for musicians compared to non-musicians (Schneider et al., 2002; Bermudez et al., 2009), and also correlated with performance in pitch processing tasks (Foster and Zatorre, 2010). These findings highlight functional and structural specialization of the auditory cortex for various acoustic features, which could underlie differences between speech and other aspects of auditory processing (Zatorre and Gandour, 2008).

Relatively few NIBS studies have examined asymmetries in the auditory cortex. As already mentioned above, several tDCS studies showed more impairment when stimulation was applied over the right compared to the left auditory cortex, in accordance with a prominent role for the right auditory cortex in pitch discrimination. In addition, Heimrath et al. (2014) showed impairment for anodal tDCS applied over the left but not the right auditory cortex for auditory temporal information processing. These tDCS findings are in line with the neuroimaging literature, since they demonstrate dissociable roles of the left and right auditory cortices for processing different type of auditory information.

These findings also highlight the need to use neuroimaging to guide NIBS applications. This consideration is especially relevant since variations in stimulus patterns (spectral and temporal) or the use of differently structured tonal patterns may differentially recruit primary and non-primary auditory cortical regions (Patterson et al., 2002), affecting cortical excitability and connectivity, and therefore impacting on NIBS outcomes. Most critically, combining NIBS with neuroimaging enables the physiological effects of the stimulation to be documented and measured, thereby allowing the researcher to determine which neural systems have been altered by the stimulation and in what way they have been modulated.

In addition, the combination of NIBS and neuroimaging enables one to assess individual differences in structural and functional organization of auditory networks, and might help to reduce the relatively high inter-subject variability of NIBS-induced changes on behavior (i.e., impairment or facilitation, or strength of the modulation). Future studies should therefore systematically assess functional organization and connectivity in order to better understand changes induced by tDCS, or any other stimulation, at a whole-brain (i.e., interconnected local and remote areas to auditory cortex) and at individual levels.

## TMS of the Auditory Cortex: Modulation of Behavior

Transcranial magnetic stimulation uses magnetic fields to induce electrical current in spatially restricted cortical regions. Compared with tDCS, TMS provides a better spatial resolution; when used over the motor cortex, it also allows the quantification of motor-evoked potentials (MEPs), a measure of the excitability of the motor system, which tDCS does not. TMS has however some disadvantages, such as evoked facial muscle twitches and loud clicking noise which may introduce confounding effects when applied over the auditory cortex, especially at high stimulation frequencies, and therefore need to be accounted for.

Similar to tDCS, TMS can have inhibitory or excitatory effects on cortical excitability, but different from tDCS, this feature is not polarity-related but rather frequency-related. Studies using TMS on the motor or visual cortices have shown that low-frequency TMS (1 Hz) decreases motor and visual cortical excitability (Chen et al., 1997; Boroojerdi et al., 2002) and high frequency TMS (>1 Hz) increases it (Pascual-Leone et al., 1994).

In addition to the frequency of stimulation, TMS effects depend also on the duration and the pattern of stimulation. For instance, single-pulse TMS delivers a single magnetic pulse and its effect is transient, whereas repetitive TMS (rTMS) delivers repeated single magnetic pulses and is able to modulate cortical activity beyond the stimulation period (Pascual-Leone et al., 1998; Klomjai et al., 2015). Paired-pulse TMS methods consist in delivering two consecutive pulses with a short inter-stimulus interval (ISI) of a few milliseconds (1–4 ms) or a long ISI (5–100 ms), and have been used to examine, respectively, intracortical inhibition or excitation (Munchau et al., 2002). The two TMS pulses can also be delivered over each hemisphere to examine inter-hemispheric inhibition (or transcallosal inhibition; Ferbert et al., 1992).

More recently, theta burst stimulation (TBS) methods have been developed based on experimental neurophysiology for inducing LTD- or LTP-like effects depending on the pattern of stimulation (Huang et al., 2005; Grossheinrich et al., 2009). TBS consists of short bursts at 50-Hz stimulation frequency and repeated at 5-Hz frequency (“theta frequency”), and neuropharmacological studies suggest that its response depends on NMDA receptor activity (Huang et al., 2007; Teo et al., 2007). Interest in using such high-frequency bursts comes from evidence of TBS application on the motor cortex, showing bi-directional and long-lasting changes on cortical excitability, such that intermittent 50-Hz bursts (iTBS) increased and continuous 50-Hz bursts (cTBS) decreased cortical excitability for up to an hour (Huang et al., 2005).

The effects of TMS parameters (frequency, duration, and pattern of bursts) on non-motor areas, such as language or auditory cortices are however still unclear. For example, Andoh et al. (2008) compared the effects of iTBS and 1-Hz rTMS applied over the temporoparietal area on an auditory discrimination task using words in native or foreign languages. The authors reported for both stimulation type decreases in response time, suggesting behavioral facilitation. A difference between the two stimulation types was found however for the discrimination of foreign words which was higher for iTBS compared with 1-Hz rTMS. The authors hypothesized frequency-dependent changes related to differences in local and remote functional connectivity with the targeted temporoparietal cortex.

Such facilitatory effects on language processing after rTMS applied over the temporoparietal cortex have been reproduced for other stimulation frequencies such as 10 or 20 Hz (Sparing et al., 2001). Differences however exist across studies regarding rTMS stimulation frequencies. For instance, Sparing et al. (2001) showed that low-frequency (1 Hz) rTMS applied over the left temporoparietal area had no effect on picture naming, whereas Andoh et al. showed facilitatory effects on a word discrimination task (Andoh et al., 2006, 2008; Andoh and Paus, 2011). Outcome variability between these studies could be related to many factors: methodological such as the procedure used to localize brain targets or the type of language task being performed. Andoh et al. (2006, 2008) and Andoh and Paus (2011) used a neuronavigation procedure to individually localize brain targets based on anatomical and functional data acquired using fMRI, whereas Sparing et al. (2001) defined “standard” brain targets using the 10-20 International System. In addition, although both Sparing’s and Andoh’s studies applied rTMS to investigate semantic processing, Sparing et al. (2001) used visual modalities, whereas Andoh et al. used auditory modalities. Differences in task modalities (visual vs. auditory) may underlie different functional organization and connectivity of the language pathway, and might differently be modulated by TMS. To overcome such issues, there is increasing interest in combining neuroimaging and TMS to investigate how functional organization and connectivity in the language pathway vary in relation to the type of modality being used.

## TMS Combined With Neuroimaging: Evidence of Asymmetry and Interhemispheric Auditory Interactions

To date, only a few studies have used functional neuroimaging to map TMS-induced effects, especially in the auditory cortex. These studies showed that after TMS applied over the auditory cortex, functional interactions occur at a large-scale level, reaching brain areas in the contralateral hemisphere (Andoh and Zatorre, 2013; Andoh et al., 2015). For instance, Andoh and Zatorre (2013) were the first to show that rTMS applied over the right auditory cortex when participants performed a melody discrimination task increased task-related neural activity in the contralateral left auditory cortex. These findings were not found for TMS applied over the left auditory cortex, suggesting specificity of the right auditory cortex for the auditory task being performed. The authors also showed that after TMS applied over the right auditory cortex, the increased activity in the contralateral left auditory cortex was related to increased interhemispheric functional connectivity between both auditory cortices. In other words, individuals with a strong functional connectivity between auditory cortices also showed increased activity in the contralateral (left) auditory cortex (Andoh and Zatorre, 2011). Moreover, the authors highlighted directionality in auditory information transfer, i.e., from the right to the left auditory cortex, likely demonstrating some auditory compensatory processes that are set up after TMS applied over the right auditory cortex. Such findings could also help to explain inter-subject variability reported in NIBS studies in basic and clinical research (Hartwigsen et al., 2013), since the degree of interhemispheric connectivity could be a predictor of TMS outcome.

Such interhemispheric interaction mechanisms have also been shown for brain areas involved in language comprehension. Andoh and Paus (2011) showed that rTMS applied over the left temporoparietal area before participants performed an auditory language comprehension task induced changes in neural activity in the right temporoparietal area. Such interhemispheric interaction processes are comparable to functional reorganization associated with recovery from language disorders (Saur et al., 2006; Hartwigsen et al., 2013). Following stroke, the brain undergoes massive plastic changes, with changes in interhemisheric inhibitory interactions between the affected and the unaffected hemisphere. NIBS therapeutic strategies have been developed to enhance “adaptive” plasticity between homologous contralateral areas via transcallosal interhemispheric inhibition processes, by stimulating either the “affected” hemisphere, or the “unaffected” hemisphere (Mansur et al., 2005; Takeuchi et al., 2005; Thiel et al., 2005, 2006; Fregni et al., 2006; Weiduschat et al., 2011).

## Evidence of Functional Asymmetry and Hemispheric Interactions in the Resting Auditory Cortex

**FIGURE 1:**
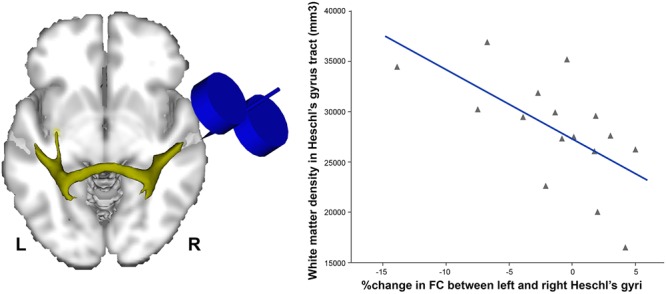
Individuals with higher density of white matter in the tract connecting both Heschl’s gyri (left) were the ones showing more decreased functional connectivity (FC) following continuous (inhibitory) TBS applied over the right Heschl’s gyrus (*r* = –0.6, *p* < 0.01). Data adapted from Andoh et al. (2015) with permission.

In order to dissociate task-related compensatory processes from TMS-induced effects, Andoh et al. (2015) applied TBS to the auditory cortex immediately before a resting-state fMRI scan, and compared it to a resting-state scan obtained prior to stimulation. Such an approach helps to reveal baseline auditory activity and connectivity, avoiding the confound of a cognitive task. Andoh et al. (2015) reported that continuous (inhibitory) TBS over applied over the right but not the left auditory cortex was related to connectivity decreases in resting-state auditory and motor networks. Interestingly, the degree of individual decreases in functional connectivity was associated with the volume of the callosal fibers connecting both auditory cortices, such that individuals with greater index of anatomical connectivity showed the greatest contralateral effects, and *vice versa* (**Figure 1**).

Although there is debate regarding structural linkage and resting-state functional connectivity (Honey et al., 2009), such findings show that asymmetry of NIBS-related effects on the auditory cortex is present at rest, and that some aspects of communication between the two auditory cortices may be directional from the right to left hemisphere, even in the absence of a task. A recent MRI diffusion-based network connectivity study using graph theory spreading activation models also showed evidence for differential patterns emanating from left vs. right auditory cortex, thereby supporting the effects seen with NIBS (Misic et al., 2018).

Functional asymmetries in the auditory cortex activity at rest were also previously reported using positron emission tomography. For instance, Geven et al. (2014) showed not only an increased “resting” metabolism in the left compared with the right primary auditory cortex but also a rightward asymmetry in the secondary and association auditory cortices. In addition, an increased resting-state functional connectivity was found between the right auditory cortex and ventral premotor areas in musically trained persons (Palomar-Garcia et al., 2017), thereby supporting the concept of enhanced coupling between auditory and motor systems as a function of musical training, and also consistent with the modulation of the resting-state motor network after auditory stimulation found in the Andoh et al. (2015)’s study. The nature of how interhemispheric connectivity is manifested, i.e., inhibitory or excitatory, is however still debated (Bloom and Hynd, 2005).

Knowledge about baseline connectivity dynamics is highly valuable since it has the potential to predict behavioral performance in perception and cognitive tasks (Sadaghiani et al., 2015; Tavor et al., 2016) and has been considered as a potential biomarker for various neurological and psychiatric diseases (Greicius, 2008; Castellanos et al., 2013; Zidda et al., 2018). For example, anomalies in connectivity between amygdala and auditory cortex have been associated with tinnitus distress (Chen et al., 2017), and disruptions in other networks such as bilateral superior frontal gyri and postcentral gyri have also been identified (Chen et al., 2016). Such findings show that resting-state MRI can reveal the activity multiple brain networks without confounding effects of cognitive ability and may be a promising translation bridge between basic and clinical research.

## Modulation of Frequency-Specific Neural Oscillations in Auditory Processing

A recent development in the field of NIBS is the use of tACS (Polania et al., 2012) and tRNS to either synchronize or desynchronize neural oscillations (Terney et al., 2008). Evidence for the potential of such techniques comes from studies showing that frequency-specific neural oscillations play a role in processing sensory information (Hong et al., 2008; Keil and Senkowski, 2018). For example, attentional modulation of cortical excitability in sensory regions has been shown to be reflected in oscillatory alpha power (∼10 Hz) under visual (Jensen and Mazaheri, 2010) or auditory tasks (Weisz et al., 2014). Therefore frequency-specific modulation of physiologically relevant brain oscillations might provide an interesting perspective into cognitive functions (Basar et al., 2001; Engel et al., 2001). Whereas in tDCS, a constant current is applied over time, tACS consist in applying a current alternating at a frequency which is believed to be associated with a particular cognitive function (Herrmann et al., 2013). In tRNS, alternating electrical currents are applied at different frequencies and amplitudes (random noise frequency spectrum; Antal and Herrmann, 2016; Heimrath et al., 2016). The mechanism underlying tRNS has been associated with stochastic resonance, which describes an enhancement of weak signals when an appropriate level of random noise is added to a non-linear system (Gingl et al., 1995)

Such techniques seem particularly relevant considering that gamma band oscillations have been associated with the processing of auditory information at the level of the auditory cortex (Rosen, 1992). Using frequency-specific 40-Hz tACS applied over bilateral auditory cortices, auditory gamma rhythm activity has been related to phoneme processing (Rufener et al., 2016b). In a second study, the same authors showed that 40-Hz tACS applied over bilateral auditory cortices improved accuracy during a phoneme categorization task in older adults (>60 years) compared with younger ones (<35), offering potential for therapy in individuals with impaired auditory temporal resolution (Rufener et al., 2016a). However, some studies also reported a large variability in the preferred frequency of the targeted cortex and it has been suggested that targeting alpha rhythm activity might be preferable since it provides clearly extractable peaks in the individual frequency spectra (Zaehle et al., 2010a; Zoefel and Davis, 2017). This is in line with findings from Neuling et al. (2012) showing that phase of alpha oscillations over the temporal cortex was related to auditory detection performance (Neuling et al., 2012).

Stimulation of auditory areas outside of the temporal cortex has recently been shown to be especially relevant for auditory working memory. Albouy et al. (2017) used magnetoencephalography to identify that theta-band activity coming from the auditory dorsal stream was higher when the task involved manipulating the stimuli in working memory (comparing tones in reversed order), compared to a simple comparison task. They then applied rhythmic TMS at the theta frequency over the left intraparietal sulcus during the silent period in between two stimuli, during which mental manipulation was occurring, and observed that behavioral performance improved significantly compared both to baseline and to an arrhythmic TMS control condition. Critically, using simultaneous electroencephalography (EEG) and TMS, they found that rhythmic TMS enhanced EEG theta power, and that the degree of enhancement predicted individual differences in degree of behavioral improvement. This study thus showed the power of combined imaging and stimulation protocols to demonstrate a causal influence of TMS on behavior, mediated by oscillatory activity in the auditory dorsal network.

## Potential of NIBS in the Developing Brain

The potential of NIBS techniques to boost sensory or cognitive functions such as auditory or language processing in a safe and non-invasive fashion motivated its use in the developing brain. The use of these neuro-enhancement techniques in pediatric populations raises however ethical concerns (Cohen Kadosh et al., 2012b; Davis, 2014; Maslen et al., 2014), particularly since data on safety and potential hazards of NIBS in children are insufficient. Indeed, differences in anatomy and function between the mature and the developing brain are not well understood; consequently, modulating the activity in the “wrong” brain area might induce abnormal patterns of activity in this area and in interconnected areas. Similarly, “boosting” capacities in certain cognitive domains might reduce functioning in others (Cohen Kadosh et al., 2012a). Although disorders related to abnormalities of auditory processing such as autism or dyslexia (Khan et al., 2011; Edgar et al., 2015) might benefit from NIBS application (Gomez et al., 2017); the use of NIBS might be still premature in developmental populations, and proper protocols still need to be established to avoid unwanted side effects.

## Short- and Long-Term Effects of NIBS on Cortical Excitability

An additional point regarding NIBS applications, particularly relevant for therapy, concerns its after-effects. The physiological bases of NIBS after-effects have not yet been clearly identified. Many arguments support the idea that the mechanisms underlying NIBS after-effects could resemble LTD and LTP described in the human auditory cortex using fMRI (Zaehle et al., 2007) or EEG (Clapp et al., 2005).

Little is known however, because most studies have examined lasting effects only on the motor cortex, and focused on the immediate effects induced after the stimulation. For instance, using tDCS over the motor cortex, perturbation in neurophysiological measures was shown to substantially outlast the stimulation period by up to 90 min (Nitsche and Paulus, 2001; Nitsche et al., 2003a). Using combined EEG and tACS, an increased in alpha band power was found lasting up to 30 min after the end of the stimulation (Zaehle et al., 2010b; Neuling et al., 2013).

Long-lasting changes in cortical excitability were also shown with tRNS, such that 10-min tRNS lasted up to 60 min (Terney et al., 2008), and 5-min tRNS lasted 10 min (Chaieb et al., 2011), highlighting relationship between NIBS parameters and their long lasting effects.

Similarly, after-effects on cortical excitability have been reported with TMS and have been related to the frequency used. Using 50-Hz bursts of TMS (e.g., cTBS), decreased cortical excitability was reported lasting up to 60 min (Huang et al., 2005). In addition, 1-Hz rTMS decreased cortical inhibition up to 30 min following the cessation of TMS (Muellbacher et al., 2000; Gerschlager et al., 2001), which was also shown by a decreased fMRI neural activity for up to 20 min (Min et al., 2016).

Effects of NIBS on auditory or language functions might not be as long lasting as for the motor cortex or more complex to assess, possibly related to ongoing cognitive processes and deserve thorough investigation using neuroplasticity markers. For instance, 20-Hz rTMS applied over the left temporal cortex induced performance facilitation lasting up to 2 min (Sparing et al., 2008). In Andoh and Zatorre (2011), using 1-Hz- or 10-Hz rTMS applied over the right auditory cortex offline (before an auditory task), changes in response time were shown to be differently modulated between time 1 (0–5.5 min) and time 2 (5.5–11 min). Such differences in behavior were related to changes in functional connectivity of auditory cortices, showing therefore relationships between after-effects of NIBS modulatory effects and underlying ongoing cognitive processes.

## Translational Approaches to Auditory Neurological Disorders

Current NIBS findings in basic research highlight inter-individual variability related to structure and function of the auditory cortex, thereby emphasizing the importance of assessing individual measures. This information is especially important for optimizing therapeutic outcome in clinical settings, e.g., tinnitus or auditory hallucinations in schizophrenia patients, since the lateralization and duration of the disorder, frequency of the occurrence of the symptoms, might affect functional organization in the auditory pathway and therefore after-effects of NIBS.

Therapeutic use of NIBS for tinnitus suppression was investigated using TMS applied over the auditory cortex. Some studies compared various rTMS protocols (e.g., 1 Hz, cTBS, and iTBS) and showed greatest reduction of tinnitus loudness for both iTBS and 1-Hz rTMS (Lorenz et al., 2010; Muller et al., 2013). The positive outcome of TMS is however short-lived. For instance, rTMS applied over auditory cortex showed tinnitus reduction for 2 s only (De Ridder et al., 2005). Other NIBS techniques (tDCS, tACS, and tRNS) have also been tested, and seem to show superiority for tRNS on tinnitus loudness and distress (Vanneste et al., 2013; Joos et al., 2015; Abtahi et al., 2018). However, differences in mechanisms of action between NIBS techniques are not well understood, and there is little knowledge about the optimal dose and interval between consecutive applications (Goldsworthy et al., 2015).

## NIBS: Future Directions

Non-invasive brain stimulation techniques such as tDCS have been a technique of choice in many clinical and research settings because it is portable, painless, and inexpensive compared with other NIBS techniques. tACS, tRNS, and tDCS have however a relatively low spatial precision, due to the large spatial distribution of the electrical current flow produced by the electrodes and is furthermore accentuated by the anatomical variability of the targeted brain structures (Parazzini et al., 2015; Alam et al., 2016). To overcome this problem, efforts have been made such as the use of high-definition or multi-electrode tDCS (Faria et al., 2011; Ruffini et al., 2014) and computational modeling of the electric field using individual structural MRI (Datta et al., 2011; Dmochowski et al., 2011; Opitz et al., 2015). These newer approaches have yet to be applied very systematically to date, but hold promise for future applications.

It is however still unclear if modulation of auditory functions will benefit from higher spatial accuracy. Present knowledge using TMS show that even targeting “accurately” the auditory cortex within a 2-cm resolution has an effect at a large scale reaching remote interconnected areas. New directions for NIBS might be therefore oriented toward “guided cortical plasticity,” consisting in combining NIBS-induced unspecific neural noise with specific behavioral training. Such approach has been successfully used to improve various perceptual or cognitive abilities in both healthy participants and in patients (Richmond et al., 2014; Looi et al., 2016).

Other NIBS techniques have also been recently developed which also hold promise for advancement into our understanding of cognitive function. For instance, transcranial pulsed current stimulation (tPCS; Jaberzadeh et al., 2014), which converts a direct current into unidirectional pulsatile current is believed to reach deep structures such as thalamus or hypothalamus (Datta et al., 2013). Moreover, transcranial ultrasound stimulation (TUS) which is based on ultrasound-induced modulation offers also a new perspective into NIBS (Tufail et al., 2011). The effects of tPCS or TUS have so far not been investigated on the auditory cortex.

## Conclusion

We highlighted the complexity of the cortical dynamics and functional interactions in the auditory cortex. We also emphasized the importance of NIBS approaches in basic science research, since they helped to better understand local and remote functional interactions in auditory areas, such as the asymmetric communications between auditory cortices. Such information is crucial and could help to optimize the application of NIBS in clinical settings. We support the combined use of NIBS with functional imaging techniques, such as fMRI or EEG/MEG to better understand the physiological consequences of stimulation of neural networks and to account for individual differences. Current findings also suggest that individual differences in structure and function could be predictive factors of NIBS outcome. Therefore, assessing functional organization and connectivity in auditory-related disorders should provide a better understanding of NIBS-induced propagation mechanisms in disease.

## Author Contributions

All authors listed have made a substantial, direct, and intellectual contribution to the work and approved it for publication.

## Conflict of Interest Statement

The authors declare that the research was conducted in the absence of any commercial or financial relationships that could be construed as a potential conflict of interest.

## References

[B1] Abtahi H. Okhovvat A. Heidari S. Gharagazarloo A. Mirdamadi M. Nilforoush M. H. (2018). Effect of transcranial direct current stimulation on short-term and long-term treatment of chronic tinnitus. *Am. J. Otolaryngol.* 39 94–96. 10.1016/j.amjoto.2018.01.001 29336898

[B2] Alam M. Truong D. Q. Khadka N. Bikson M. (2016). Spatial and polarity precision of concentric high-definition transcranial direct current stimulation (HD-tDCS). *Phys. Med. Biol.* 61 4506–4521. 10.1088/0031-9155/61/12/4506 27223853

[B3] Albouy P. Weiss A. Baillet S. Zatorre R. J. (2017). Selective entrainment of theta oscillations in the dorsal stream causally enhances auditory working memory performance. *Neuron* 94:e195. 10.1016/j.neuron.2017.03.015 28343866

[B4] Andoh J. Artiges E. Pallier C. Riviere D. Mangin J. F. Cachia A. (2006). Modulation of language areas with functional MR image-guided magnetic stimulation. *Neuroimage* 29 619–627. 10.1016/j.neuroimage.2005.07.029 16168674

[B5] Andoh J. Artiges E. Pallier C. Riviere D. Mangin J. F. Paillere-Martinot M. L. (2008). Priming frequencies of transcranial magnetic stimulation over Wernicke’s area modulate word detection. *Cereb. Cortex* 18 210–216. 10.1093/cercor/bhm047 17490990

[B6] Andoh J. Matsushita R. Zatorre R. J. (2015). Asymmetric interhemispheric transfer in the auditory network: evidence from TMS, resting-state fMRI, and diffusion imaging. *J. Neurosci.* 35 14602–14611. 10.1523/JNEUROSCI.2333-15.2015 26511249 PMC6605461

[B7] Andoh J. Paus T. (2011). Combining functional neuroimaging with off-line brain stimulation: modulation of task-related activity in language areas. *J. Cogn. Neurosci.* 23 349–361. 10.1162/jocn.2010.21449 20146606

[B8] Andoh J. Zatorre R. J. (2011). Interhemispheric connectivity influences the degree of modulation of TMS-induced effects during auditory processing. *Front. Psychol.* 2:161. 10.3389/fpsyg.2011.00161 21811478 PMC3139954

[B9] Andoh J. Zatorre R. J. (2013). Mapping interhemispheric connectivity using functional MRI after transcranial magnetic stimulation on the human auditory cortex. *Neuroimage* 79 162–171. 10.1016/j.neuroimage.2013.04.078 23631993

[B10] Antal A. Herrmann C. S. (2016). Transcranial alternating current and random noise stimulation: possible mechanisms. *Neural Plast.* 2016:3616807. 10.1155/2016/3616807 27242932 PMC4868897

[B11] Antal A. Nitsche M. A. Kincses T. Z. Kruse W. Hoffmann K. P. Paulus W. (2004). Facilitation of visuo-motor learning by transcranial direct current stimulation of the motor and extrastriate visual areas in humans. *Eur. J. Neurosci.* 19 2888–2892. 10.1111/j.1460-9568.2004.03367.x 15147322

[B12] Basar E. Basar-Eroglu C. Karakas S. Schurmann M. (2001). Gamma, alpha, delta, and theta oscillations govern cognitive processes. *Int. J. Psychophysiol.* 39 241–248. 10.1016/S0167-8760(00)00145-8 11163901

[B13] Batsikadze G. Moliadze V. Paulus W. Kuo M. F. Nitsche M. A. (2013). Partially non-linear stimulation intensity-dependent effects of direct current stimulation on motor cortex excitability in humans. *J. Physiol.* 591 1987–2000. 10.1113/jphysiol.2012.249730 23339180 PMC3624864

[B14] Belin P. Zilbovicius M. Crozier S. Thivard L. Fontaine A. Masure M. C. (1998). Lateralization of speech and auditory temporal processing. *J. Cogn. Neurosci.* 10 536–540. 10.1162/089892998562834 9712682

[B15] Bermudez P. Lerch J. P. Evans A. C. Zatorre R. J. (2009). Neuroanatomical correlates of musicianship as revealed by cortical thickness and voxel-based morphometry. *Cereb. Cortex* 19 1583–1596. 10.1093/cercor/bhn196 19073623

[B16] Bindman L. J. Lippold O. C. Redfearn J. W. (1964). The action of brief polarizing currents on the cerebral cortex of the rat (1) during current flow and (2) in the production of long-lasting after-effects. *J. Physiol.* 172 369–382. 10.1113/jphysiol.1964.sp007425 14199369 PMC1368854

[B17] Bloom J. S. Hynd G. W. (2005). The role of the corpus callosum in interhemispheric transfer of information: excitation or inhibition? *Neuropsychol. Rev.* 15 59–71. 10.1007/s11065-005-6252-y 16211466

[B18] Boroojerdi B. Meister I. G. Foltys H. Sparing R. Cohen L. G. Topper R. (2002). Visual and motor cortex excitability: a transcranial magnetic stimulation study. *Clin. Neurophysiol.* 113 1501–1504. 10.1016/S1388-2457(02)00198-0 12169333

[B19] Castellanos F. X. Di Martino A. Craddock R. C. Mehta A. D. Milham M. P. (2013). Clinical applications of the functional connectome. *Neuroimage* 80 527–540. 10.1016/j.neuroimage.2013.04.083 23631991 PMC3809093

[B20] Chaieb L. Paulus W. Antal A. (2011). Evaluating aftereffects of short-duration transcranial random noise stimulation on cortical excitability. *Neural Plast.* 2011:105927. 10.1155/2011/105927 21808744 PMC3144676

[B21] Chen R. Classen J. Gerloff C. Celnik P. Wassermann E. M. Hallett M. (1997). Depression of motor cortex excitability by low-frequency transcranial magnetic stimulation. *Neurology* 48 1398–1403. 10.1212/WNL.48.5.1398 9153480

[B22] Chen Y. C. Feng Y. Xu J. J. Mao C. N. Xia W. Ren J. (2016). Disrupted brain functional network architecture in chronic tinnitus patients. *Front. Aging Neurosci.* 8:174. 10.3389/fnagi.2016.00174 27458377 PMC4937025

[B23] Chen Y. C. Xia W. Chen H. Feng Y. Xu J. J. Gu J. P. (2017). Tinnitus distress is linked to enhanced resting-state functional connectivity from the limbic system to the auditory cortex. *Hum. Brain Mapp.* 38 2384–2397. 10.1002/hbm.23525 28112466 PMC6866871

[B24] Clapp W. C. Kirk I. J. Hamm J. P. Shepherd D. Teyler T. J. (2005). Induction of LTP in the human auditory cortex by sensory stimulation. *Eur. J. Neurosci.* 22 1135–1140. 10.1111/j.1460-9568.2005.04293.x 16176355

[B25] Cohen Kadosh R. Gertner L. Terhune D. B. (2012a). Exceptional abilities in the spatial representation of numbers and time: insights from synesthesia. *Neuroscientist* 18 208–215. 10.1177/1073858411402835 21571722

[B26] Cohen Kadosh R. Levy N. O’shea J. Shea N. Savulescu J. (2012b). The neuroethics of non-invasive brain stimulation. *Curr. Biol.* 22 R108–R111. 10.1016/j.cub.2012.01.013 22361141 PMC4347660

[B27] Cohen Kadosh R. Soskic S. Iuculano T. Kanai R. Walsh V. (2010). Modulating neuronal activity produces specific and long-lasting changes in numerical competence. *Curr. Biol.* 20 2016–2020. 10.1016/j.cub.2010.10.007 21055945 PMC2990865

[B28] DaSilva A. F. Volz M. S. Bikson M. Fregni F. (2011). Electrode positioning and montage in transcranial direct current stimulation. *J. Vis. Exp.* 51:2744. 10.3791/2744 21654618 PMC3339846

[B29] Datta A. Baker J. M. Bikson M. Fridriksson J. (2011). Individualized model predicts brain current flow during transcranial direct-current stimulation treatment in responsive stroke patient. *Brain Stimul.* 4 169–174. 10.1016/j.brs.2010.11.001 21777878 PMC3142347

[B30] Datta A. Dmochowski J. P. Guleyupoglu B. Bikson M. Fregni F. (2013). Cranial electrotherapy stimulation and transcranial pulsed current stimulation: a computer based high-resolution modeling study. *Neuroimage* 65 280–287. 10.1016/j.neuroimage.2012.09.062 23041337

[B31] Davis N. J. (2014). Transcranial stimulation of the developing brain: a plea for extreme caution. *Front. Hum. Neurosci.* 8:600. 10.3389/fnhum.2014.00600 25140146 PMC4122183

[B32] De Ridder D. Verstraeten E. Van Der Kelen K. De Mulder G. Sunaert S. Verlooy J. (2005). Transcranial magnetic stimulation for tinnitus: influence of tinnitus duration on stimulation parameter choice and maximal tinnitus suppression. *Otol. Neurotol.* 26 616–619. 10.1097/01.mao.0000178146.91139.3c 16015156

[B33] Dehaene-Lambertz G. Hertz-Pannier L. Dubois J. (2006). Nature and nurture in language acquisition: anatomical and functional brain-imaging studies in infants. *Trends Neurosci.* 29 367–373. 10.1016/j.tins.2006.05.011 16815562

[B34] Dmochowski J. P. Datta A. Bikson M. Su Y. Parra L. C. (2011). Optimized multi-electrode stimulation increases focality and intensity at target. *J. Neural Eng.* 8:046011. 10.1088/1741-2560/8/4/046011 21659696

[B35] Edgar J. C. Fisk Iv C. L. Berman J. I. Chudnovskaya D. Liu S. Pandey J. (2015). Auditory encoding abnormalities in children with autism spectrum disorder suggest delayed development of auditory cortex. *Mol. Autism* 6:69. 10.1186/s13229-015-0065-5 26719787 PMC4696177

[B36] Engel A. K. Fries P. Singer W. (2001). Dynamic predictions: oscillations and synchrony in top-down processing. *Nat. Rev. Neurosci.* 2 704–716. 10.1038/35094565 11584308

[B37] Faria P. Hallett M. Miranda P. C. (2011). A finite element analysis of the effect of electrode area and inter-electrode distance on the spatial distribution of the current density in tDCS. *J Neural Eng.* 8:066017. 10.1088/1741-2560/8/6/066017 22086257 PMC3411515

[B38] Ferbert A. Priori A. Rothwell J. C. Day B. L. Colebatch J. G. Marsden C. D. (1992). Interhemispheric inhibition of the human motor cortex. *J. Physiol.* 453 525–546. 10.1113/jphysiol.1992.sp019243 1464843 PMC1175572

[B39] Filmer H. L. Dux P. E. Mattingley J. B. (2014). Applications of transcranial direct current stimulation for understanding brain function. *Trends Neurosci.* 37 742–753. 10.1016/j.tins.2014.08.003 25189102

[B40] Foster N. E. Zatorre R. J. (2010). Cortical structure predicts success in performing musical transformation judgments. *Neuroimage* 53 26–36. 10.1016/j.neuroimage.2010.06.042 20600982

[B41] Fregni F. Boggio P. S. Valle A. C. Rocha R. R. Duarte J. Ferreira M. J. (2006). A sham-controlled trial of a 5-day course of repetitive transcranial magnetic stimulation of the unaffected hemisphere in stroke patients. *Stroke* 37 2115–2122. 10.1161/01.STR.0000231390.58967.6b 16809569

[B42] Fritsch B. Reis J. Martinowich K. Schambra H. M. Ji Y. Cohen L. G. (2010). Direct current stimulation promotes BDNF-dependent synaptic plasticity: potential implications for motor learning. *Neuron* 66 198–204. 10.1016/j.neuron.2010.03.035 20434997 PMC2864780

[B43] Gerschlager W. Siebner H. R. Rothwell J. C. (2001). Decreased corticospinal excitability after subthreshold 1 Hz rTMS over lateral premotor cortex. *Neurology* 57 449–455. 10.1212/WNL.57.3.449 11502912

[B44] Geven L. I. De Kleine E. Willemsen A. T. Van Dijk P. (2014). Asymmetry in primary auditory cortex activity in tinnitus patients and controls. *Neuroscience* 256 117–125. 10.1016/j.neuroscience.2013.10.015 24161276

[B45] Gingl Z. Kiss L. Moss F. (1995). Non-dynamical stochastic resonance: theory and experiments with white and arbitrarily coloured noise. *Europhys. Lett.* 29:191 10.1209/0295-5075/29/3/001

[B46] Goldsworthy M. R. Pitcher J. B. Ridding M. C. (2015). Spaced noninvasive brain stimulation: prospects for inducing long-lasting human cortical plasticity. *Neurorehabil. Neural Repair* 29 714–721. 10.1177/1545968314562649 25505220

[B47] Gomez L. Vidal B. Maragoto C. Morales L. M. Berrillo S. Vera Cuesta H. (2017). Non-invasive brain stimulation for children with autism spectrum disorders: a short-term outcome study. *Behav. Sci. (Basel.)* 7:63. 10.3390/bs7030063 28926975 PMC5618071

[B48] Greicius M. (2008). Resting-state functional connectivity in neuropsychiatric disorders. *Curr. Opin. Neurol.* 21 424–430. 10.1097/WCO.0b013e328306f2c5 18607202

[B49] Grossheinrich N. Rau A. Pogarell O. Hennig-Fast K. Reinl M. Karch S. (2009). Theta burst stimulation of the prefrontal cortex: safety and impact on cognition, mood, and resting electroencephalogram. *Biol. Psychiatry* 65 778–784. 10.1016/j.biopsych.2008.10.029 19070834

[B50] Hartwigsen G. Saur D. Price C. J. Ulmer S. Baumgaertner A. Siebner H. R. (2013). Perturbation of the left inferior frontal gyrus triggers adaptive plasticity in the right homologous area during speech production. *Proc. Natl. Acad. Sci. U.S.A.* 110 16402–16407. 10.1073/pnas.1310190110 24062469 PMC3799383

[B51] Heimrath K. Fiene M. Rufener K. S. Zaehle T. (2016). Modulating human auditory processing by transcranial electrical stimulation. *Front. Cell Neurosci.* 10:53 10.3389/fncel.2016.00053 PMC4779894 27013969

[B52] Heimrath K. Kuehne M. Heinze H. J. Zaehle T. (2014). Transcranial direct current stimulation (tDCS) traces the predominance of the left auditory cortex for processing of rapidly changing acoustic information. *Neuroscience* 261 68–73. 10.1016/j.neuroscience.2013.12.031 24374325

[B53] Herrmann C. S. Rach S. Neuling T. Struber D. (2013). Transcranial alternating current stimulation: a review of the underlying mechanisms and modulation of cognitive processes. *Front. Hum. Neurosci.* 7:279. 10.3389/fnhum.2013.00279 23785325 PMC3682121

[B54] Honey C. J. Sporns O. Cammoun L. Gigandet X. Thiran J. P. Meuli R. (2009). Predicting human resting-state functional connectivity from structural connectivity. *Proc. Natl. Acad. Sci. U.S.A.* 106 2035–2040. 10.1073/pnas.0811168106 19188601 PMC2634800

[B55] Hong L. E. Buchanan R. W. Thaker G. K. Shepard P. D. Summerfelt A. (2008). Beta (∼16 Hz) frequency neural oscillations mediate auditory sensory gating in humans. *Psychophysiology* 45 197–204. 10.1111/j.1469-8986.2007.00624.x 17995907

[B56] Huang Y. Z. Chen R. S. Rothwell J. C. Wen H. Y. (2007). The after-effect of human theta burst stimulation is NMDA receptor dependent. *Clin. Neurophysiol.* 118 1028–1032. 10.1016/j.clinph.2007.01.021 17368094

[B57] Huang Y. Z. Edwards M. J. Rounis E. Bhatia K. P. Rothwell J. C. (2005). Theta burst stimulation of the human motor cortex. *Neuron* 45 201–206. 10.1016/j.neuron.2004.12.033 15664172

[B58] Iuculano T. Cohen Kadosh R. (2013). The mental cost of cognitive enhancement. *J. Neurosci.* 33 4482–4486. 10.1523/JNEUROSCI.4927-12.2013 23467363 PMC3672974

[B59] Jaberzadeh S. Bastani A. Zoghi M. (2014). Anodal transcranial pulsed current stimulation: a novel technique to enhance corticospinal excitability. *Clin. Neurophysiol.* 125 344–351. 10.1016/j.clinph.2013.08.025 24074626

[B60] Jensen O. Mazaheri A. (2010). Shaping functional architecture by oscillatory alpha activity: gating by inhibition. *Front. Hum. Neurosci.* 4:186. 10.3389/fnhum.2010.00186 21119777 PMC2990626

[B61] Joos K. De Ridder D. Vanneste S. (2015). The differential effect of low- versus high-frequency random noise stimulation in the treatment of tinnitus. *Exp. Brain Res.* 233 1433–1440. 10.1007/s00221-015-4217-9 25694243

[B62] Keil J. Senkowski D. (2018). Neural oscillations orchestrate multisensory processing. *Neuroscientist* 10.1177/1073858418755352 [Epub ahead of print]. 29424265

[B63] Khan A. Hamalainen J. A. Leppanen P. H. Lyytinen H. (2011). Auditory event-related potentials show altered hemispheric responses in dyslexia. *Neurosci. Lett.* 498 127–132. 10.1016/j.neulet.2011.04.074 21565249

[B64] Klomjai W. Katz R. Lackmy-Vallee A. (2015). Basic principles of transcranial magnetic stimulation (TMS) and repetitive TMS (rTMS). *Ann. Phys. Rehabil. Med.* 58 208–213. 10.1016/j.rehab.2015.05.005 26319963

[B65] Looi C. Y. Duta M. Brem A. K. Huber S. Nuerk H. C. Cohen Kadosh R. (2016). Combining brain stimulation and video game to promote long-term transfer of learning and cognitive enhancement. *Sci. Rep.* 6:22003. 10.1038/srep22003 26902664 PMC4763231

[B66] Lorenz I. Muller N. Schlee W. Langguth B. Weisz N. (2010). Short-term effects of single repetitive TMS sessions on auditory evoked activity in patients with chronic tinnitus. *J. Neurophysiol.* 104 1497–1505. 10.1152/jn.00370.2010 20592125

[B67] Mansur C. G. Fregni F. Boggio P. S. Riberto M. Gallucci-Neto J. Santos C. M. (2005). A sham stimulation-controlled trial of rTMS of the unaffected hemisphere in stroke patients. *Neurology* 64 1802–1804. 10.1212/01.WNL.0000161839.38079.92 15911819

[B68] Maslen H. Earp B. D. Cohen Kadosh R. Savulescu J. (2014). Brain stimulation for treatment and enhancement in children: an ethical analysis. *Front. Hum. Neurosci.* 8:953. 10.3389/fnhum.2014.00953 25566011 PMC4270184

[B69] Mathys C. Loui P. Zheng X. Schlaug G. (2010). Non-invasive brain stimulation applied to Heschl’s gyrus modulates pitch discrimination. *Front. Psychol.* 1:193. 10.3389/fpsyg.2010.00193 21286253 PMC3028589

[B70] Matsushita R. Andoh J. Zatorre R. J. (2015). Polarity-specific transcranial direct current stimulation disrupts auditory pitch learning. *Front. Neurosci.* 9:174. 10.3389/fnins.2015.00174 26041982 PMC4434966

[B71] Meyer M. Liem F. Hirsiger S. Jancke L. Hanggi J. (2014). Cortical surface area and cortical thickness demonstrate differential structural asymmetry in auditory-related areas of the human cortex. *Cereb. Cortex* 24 2541–2552. 10.1093/cercor/bht094 23645712

[B72] Min Y. S. Park J. W. Jin S. U. Jang K. E. Lee B. J. Lee H. J. (2016). Neuromodulatory effects of offline low-frequency repetitive transcranial magnetic stimulation of the motor cortex: a functional magnetic resonance imaging study. *Sci. Rep.* 6:36058. 10.1038/srep36058 27786301 PMC5081540

[B73] Misic B. Betzel R. F. Griffa A. De Reus M. A. He Y. Zuo X. N. (2018). Network-based asymmetry of the human auditory system. *Cereb. Cortex* 28 2655–2664. 10.1093/cercor/bhy101 29722805 PMC5998951

[B74] Monte-Silva K. Kuo M. F. Hessenthaler S. Fresnoza S. Liebetanz D. Paulus W. (2013). Induction of late LTP-like plasticity in the human motor cortex by repeated non-invasive brain stimulation. *Brain Stimul.* 6 424–432. 10.1016/j.brs.2012.04.011 22695026

[B75] Muellbacher W. Ziemann U. Boroojerdi B. Hallett M. (2000). Effects of low-frequency transcranial magnetic stimulation on motor excitability and basic motor behavior. *Clin. Neurophysiol.* 111 1002–1007. 10.1016/S1388-2457(00)00284-4 10825706

[B76] Muller N. Lorenz I. Langguth B. Weisz N. (2013). rTMS induced tinnitus relief is related to an increase in auditory cortical alpha activity. *PLoS One* 8:e55557. 10.1371/journal.pone.0055557 23390539 PMC3563643

[B77] Munchau A. Bloem B. R. Irlbacher K. Trimble M. R. Rothwell J. C. (2002). Functional connectivity of human premotor and motor cortex explored with repetitive transcranial magnetic stimulation. *J. Neurosci.* 22 554–561. 10.1523/JNEUROSCI.22-02-00554.2002 11784802 PMC6758651

[B78] Neuling T. Rach S. Herrmann C. S. (2013). Orchestrating neuronal networks: sustained after-effects of transcranial alternating current stimulation depend upon brain states. *Front. Hum. Neurosci.* 7:161. 10.3389/fnhum.2013.00161 23641206 PMC3639376

[B79] Neuling T. Rach S. Wagner S. Wolters C. H. Herrmann C. S. (2012). Good vibrations: oscillatory phase shapes perception. *Neuroimage* 63 771–778. 10.1016/j.neuroimage.2012.07.024 22836177

[B80] Nitsche M. A. Cohen L. G. Wassermann E. M. Priori A. Lang N. Antal A. (2008). Transcranial direct current stimulation: state of the art 2008. *Brain Stimul.* 1 206–223. 10.1016/j.brs.2008.06.004 20633386

[B81] Nitsche M. A. Liebetanz D. Antal A. Lang N. Tergau F. Paulus W. (2003a). Modulation of cortical excitability by weak direct current stimulation–technical, safety and functional aspects. *Suppl. Clin. Neurophysiol.* 56 255–276. 10.1016/S1567-424X(09)70230-2 14677403

[B82] Nitsche M. A. Nitsche M. S. Klein C. C. Tergau F. Rothwell J. C. Paulus W. (2003b). Level of action of cathodal DC polarisation induced inhibition of the human motor cortex. *Clin. Neurophysiol.* 114 600–604. 12686268 10.1016/s1388-2457(02)00412-1

[B83] Nitsche M. A. Paulus W. (2000). Excitability changes induced in the human motor cortex by weak transcranial direct current stimulation. *J. Physiol.* 527(Pt 3) 633–639. 10.1111/j.1469-7793.2000.t01-1-00633.x 10990547 PMC2270099

[B84] Nitsche M. A. Paulus W. (2001). Sustained excitability elevations induced by transcranial DC motor cortex stimulation in humans. *Neurology* 57 1899–1901. 10.1212/WNL.57.10.1899 11723286

[B85] Opitz A. Paulus W. Will S. Antunes A. Thielscher A. (2015). Determinants of the electric field during transcranial direct current stimulation. *Neuroimage* 109 140–150. 10.1016/j.neuroimage.2015.01.033 25613437

[B86] Palomar-Garcia M. A. Zatorre R. J. Ventura-Campos N. Bueicheku E. Avila C. (2017). Modulation of functional connectivity in auditory-motor networks in musicians compared with nonmusicians. *Cereb. Cortex* 27 2768–2778. 10.1093/cercor/bhw120 27166170

[B87] Parazzini M. Fiocchi S. Liorni I. Ravazzani P. (2015). Effect of the interindividual variability on computational modeling of transcranial direct current stimulation. *Comput. Intell. Neurosci.* 2015:963293. 10.1155/2015/963293 26265912 PMC4523656

[B88] Pascual-Leone A. Tormos J. M. Keenan J. Tarazona F. Canete C. Catala M. D. (1998). Study and modulation of human cortical excitability with transcranial magnetic stimulation. *J. Clin. Neurophysiol.* 15 333–343. 10.1097/00004691-199807000-00005 9736467

[B89] Pascual-Leone A. Valls-Sole J. Wassermann E. M. Hallett M. (1994). Responses to rapid-rate transcranial magnetic stimulation of the human motor cortex. *Brain* 117(Pt 4) 847–858. 10.1093/brain/117.4.847 7922470

[B90] Patterson R. D. Uppenkamp S. Johnsrude I. S. Griffiths T. D. (2002). The processing of temporal pitch and melody information in auditory cortex. *Neuron* 36 767–776. 10.1016/S0896-6273(02)01060-7 12441063

[B91] Penhune V. B. Zatorre R. J. Macdonald J. D. Evans A. C. (1996). Interhemispheric anatomical differences in human primary auditory cortex: probabilistic mapping and volume measurement from magnetic resonance scans. *Cereb. Cortex* 6 661–672. 10.1093/cercor/6.5.661 8921202

[B92] Poeppel D. A. (2003). The analysis of speech in different temporal integration windows: cerebral lateralization as ’asymmetric sampling in time. *J. Speech Commun.* 41 245–255. 10.1016/S0167-6393(02)00107-3 19162052

[B93] Polania R. Nitsche M. A. Korman C. Batsikadze G. Paulus W. (2012). The importance of timing in segregated theta phase-coupling for cognitive performance. *Curr. Biol.* 22 1314–1318. 10.1016/j.cub.2012.05.021 22683259

[B94] Richmond L. L. Wolk D. Chein J. Olson I. R. (2014). Transcranial direct current stimulation enhances verbal working memory training performance over time and near transfer outcomes. *J. Cogn. Neurosci.* 26 2443–2454. 10.1162/jocn_a_00657 24742190

[B95] Rosen S. (1992). Temporal information in speech: acoustic, auditory and linguistic aspects. *Philos. Trans. R. Soc. Lond. B Biol. Sci.* 336 367–373. 10.1098/rstb.1992.0070 1354376

[B96] Rufener K. S. Oechslin M. S. Zaehle T. Meyer M. (2016a). Transcranial alternating current stimulation (tACS) differentially modulates speech perception in young and older adults. *Brain Stimul.* 9 560–565. 10.1016/j.brs.2016.04.002 27157057

[B97] Rufener K. S. Zaehle T. Oechslin M. S. Meyer M. (2016b). 40Hz-Transcranial alternating current stimulation (tACS) selectively modulates speech perception. *Int. J. Psychophysiol.* 101 18–24. 10.1016/j.ijpsycho.2016.01.002 26779822

[B98] Ruffini G. Fox M. D. Ripolles O. Miranda P. C. Pascual-Leone A. (2014). Optimization of multifocal transcranial current stimulation for weighted cortical pattern targeting from realistic modeling of electric fields. *Neuroimage* 89 216–225. 10.1016/j.neuroimage.2013.12.002 24345389 PMC3944133

[B99] Sadaghiani S. Poline J. B. Kleinschmidt A. D’esposito M. (2015). Ongoing dynamics in large-scale functional connectivity predict perception. *Proc. Natl. Acad. Sci. U.S.A.* 112 8463–8468. 10.1073/pnas.1420687112 26106164 PMC4500238

[B100] Saur D. Lange R. Baumgaertner A. Schraknepper V. Willmes K. Rijntjes M. (2006). Dynamics of language reorganization after stroke. *Brain* 129 1371–1384. 10.1093/brain/awl090 16638796

[B101] Schneider P. Scherg M. Dosch H. G. Specht H. J. Gutschalk A. Rupp A. (2002). Morphology of Heschl’s gyrus reflects enhanced activation in the auditory cortex of musicians. *Nat. Neurosci.* 5 688–694. 10.1038/nn871 12068300

[B102] Schonwiesner M. Rubsamen R. Von Cramon D. Y. (2005). Hemispheric asymmetry for spectral and temporal processing in the human antero-lateral auditory belt cortex. *Eur. J. Neurosci.* 22 1521–1528. 10.1111/j.1460-9568.2005.04315.x 16190905

[B103] Sparing R. Buelte D. Meister I. G. Paus T. Fink G. R. (2008). Transcranial magnetic stimulation and the challenge of coil placement: a comparison of conventional and stereotaxic neuronavigational strategies. *Hum. Brain Mapp.* 29 82–96. 10.1002/hbm.20360 17318831 PMC6871049

[B104] Sparing R. Mottaghy F. M. Hungs M. Brugmann M. Foltys H. Huber W. (2001). Repetitive transcranial magnetic stimulation effects on language function depend on the stimulation parameters. *J. Clin. Neurophysiol.* 18 326–330. 10.1097/00004691-200107000-00004 11673698

[B105] Stagg C. J. Jayaram G. Pastor D. Kincses Z. T. Matthews P. M. Johansen-Berg H. (2011). Polarity and timing-dependent effects of transcranial direct current stimulation in explicit motor learning. *Neuropsychologia* 49 800–804. 10.1016/j.neuropsychologia.2011.02.009 21335013 PMC3083512

[B106] Takeuchi N. Chuma T. Matsuo Y. Watanabe I. Ikoma K. (2005). Repetitive transcranial magnetic stimulation of contralesional primary motor cortex improves hand function after stroke. *Stroke* 36 2681–2686. 10.1161/01.STR.0000189658.51972.34 16254224

[B107] Tang M. F. Hammond G. R. (2013). Anodal transcranial direct current stimulation over auditory cortex degrades frequency discrimination by affecting temporal, but not place, coding. *Eur. J. Neurosci.* 38 2802–2811. 10.1111/ejn.12280 23763771

[B108] Tavor I. Parker Jones O. Mars R. B. Smith S. M. Behrens T. E. Jbabdi S. (2016). Task-free MRI predicts individual differences in brain activity during task performance. *Science* 352 216–220. 10.1126/science.aad8127 27124457 PMC6309730

[B109] Teo J. T. Swayne O. B. Rothwell J. C. (2007). Further evidence for NMDA-dependence of the after-effects of human theta burst stimulation. *Clin. Neurophysiol.* 118 1649–1651. 10.1016/j.clinph.2007.04.010 17502166

[B110] Terney D. Chaieb L. Moliadze V. Antal A. Paulus W. (2008). Increasing human brain excitability by transcranial high-frequency random noise stimulation. *J. Neurosci.* 28 14147–14155. 10.1523/JNEUROSCI.4248-08.2008 19109497 PMC6671476

[B111] Thiel A. Habedank B. Herholz K. Kessler J. Winhuisen L. Haupt W. F. (2006). From the left to the right: How the brain compensates progressive loss of language function. *Brain Lang.* 98 57–65. 10.1016/j.bandl.2006.01.007 16519926

[B112] Thiel A. Haupt W. F. Habedank B. Winhuisen L. Herholz K. Kessler J. (2005). Neuroimaging-guided rTMS of the left inferior frontal gyrus interferes with repetition priming. *Neuroimage* 25 815–823. 10.1016/j.neuroimage.2004.12.028 15808982

[B113] Tufail Y. Yoshihiro A. Pati S. Li M. M. Tyler W. J. (2011). Ultrasonic neuromodulation by brain stimulation with transcranial ultrasound. *Nat. Protoc.* 6 1453–1470. 10.1038/nprot.2011.371 21886108

[B114] Vanneste S. Fregni F. De Ridder D. (2013). Head-to-head comparison of transcranial random noise stimulation, transcranial AC stimulation, and transcranial DC stimulation for tinnitus. *Front. Psychiatry* 4:158. 10.3389/fpsyt.2013.00158 24391599 PMC3866637

[B115] Warrier C. Wong P. Penhune V. Zatorre R. Parrish T. Abrams D. (2009). Relating structure to function: Heschl’s gyrus and acoustic processing. *J. Neurosci.* 29 61–69. 10.1523/JNEUROSCI.3489-08.2009 19129385 PMC3341414

[B116] Weiduschat N. Thiel A. Rubi-Fessen I. Hartmann A. Kessler J. Merl P. (2011). Effects of repetitive transcranial magnetic stimulation in aphasic stroke: a randomized controlled pilot study. *Stroke* 42 409–415. 10.1161/STROKEAHA.110.597864 21164121

[B117] Weisz N. Muller N. Jatzev S. Bertrand O. (2014). Oscillatory alpha modulations in right auditory regions reflect the validity of acoustic cues in an auditory spatial attention task. *Cereb. Cortex* 24 2579–2590. 10.1093/cercor/bht113 23645711

[B118] Zaehle T. Beretta M. Jäncke L. Herrmann C. S. Sandmann P. (2011). Excitability changes induced in the human auditory cortex by transcranial direct current stimulation: direct electrophysiological evidence. *Exp. Brain. Res.* 215 135–140. 10.1007/s00221-011-2879-5 21964868

[B119] Zaehle T. Clapp W. C. Hamm J. P. Meyer M. Kirk I. J. (2007). Induction of LTP-like changes in human auditory cortex by rapid auditory stimulation: an FMRI study. *Restor. Neurol. Neurosci.* 25 251–259. 17943003

[B120] Zaehle T. Jancke L. Herrmann C. S. Meyer M. (2009). Pre-attentive spectro-temporal feature processing in the human auditory system. *Brain Topogr.* 22 97–108. 10.1007/s10548-009-0085-6 19266276

[B121] Zaehle T. Lenz D. Ohl F. W. Herrmann C. S. (2010a). Resonance phenomena in the human auditory cortex: individual resonance frequencies of the cerebral cortex determine electrophysiological responses. *Exp. Brain Res.* 203 629–635. 10.1007/s00221-010-2265-8 20449728

[B122] Zaehle T. Rach S. Herrmann C. S. (2010b). Transcranial alternating current stimulation enhances individual alpha activity in human EEG. *PLoS One* 5:e13766. 10.1371/journal.pone.0013766 21072168 PMC2967471

[B123] Zaehle T. Wustenberg T. Meyer M. Jancke L. (2004). Evidence for rapid auditory perception as the foundation of speech processing: a sparse temporal sampling fMRI study. *Eur. J. Neurosci.* 20 2447–2456. 10.1111/j.1460-9568.2004.03687.x 15525285

[B124] Zatorre R. J. Belin P. (2001). Spectral and temporal processing in human auditory cortex. *Cereb. Cortex* 11 946–953. 10.1093/cercor/11.10.946 11549617

[B125] Zatorre R. J. Gandour J. T. (2008). Neural specializations for speech and pitch: moving beyond the dichotomies. *Philos. Trans. R. Soc. B Biol. Sci.* 363 1087–1104. 10.1098/rstb.2007.2161 17890188 PMC2606798

[B126] Zidda F. Andoh J. Pohlack S. Winkelmann T. Dinu-Biringer R. Cavalli J. (2018). Default mode network connectivity of fear- and anxiety-related cue and context conditioning. *Neuroimage* 165 190–199. 10.1016/j.neuroimage.2017.10.024 29050910

[B127] Zoefel B. Davis M. H. (2017). Transcranial electric stimulation for the investigation of speech perception and comprehension. *Lang. Cogn. Neurosci.* 32 910–923. 10.1080/23273798.2016.1247970 28670598 PMC5470108

